# Associations Between Physiological Signals Captured Using Wearable Sensors and Self-reported Outcomes Among Adults in Alcohol Use Disorder Recovery: Development and Usability Study

**DOI:** 10.2196/27891

**Published:** 2021-07-21

**Authors:** Parastoo Alinia, Ramesh Kumar Sah, Michael McDonell, Patricia Pendry, Sara Parent, Hassan Ghasemzadeh, Michael John Cleveland

**Affiliations:** 1 School of Electrical Engineering and Computer Science Washington State University Pullman, WA United States; 2 Elson S. Floyd College of Medicine Washington State University Pullman, WA United States; 3 Department of Human Development Washington State University Pullman, WA United States

**Keywords:** alcohol relapse prevention, stress markers, alcohol consumption, electrodermal activity, heart rate variability, emotion, mobile phone

## Abstract

**Background:**

Previous research has highlighted the role of stress in substance misuse and addiction, particularly for relapse risk. Mobile health interventions that incorporate real-time monitoring of physiological markers of stress offer promise for delivering tailored interventions to individuals during high-risk states of heightened stress to prevent alcohol relapse. Before such interventions can be developed, measurements of these processes in ambulatory, real-world settings are needed.

**Objective:**

This research is a proof-of-concept study to establish the feasibility of using a wearable sensor device to continuously monitor stress in an ambulatory setting. Toward that end, we first aimed to examine the quality of 2 continuously monitored physiological signals—electrodermal activity (EDA) and heart rate variability (HRV)—and show that the data follow standard quality measures according to the literature. Next, we examined the associations between the statistical features extracted from the EDA and HRV signals and self-reported outcomes.

**Methods:**

Participants (N=11; female: n=10) were asked to wear an Empatica E4 wearable sensor for continuous unobtrusive physiological signal collection for up to 14 days. During the same time frame, participants responded to a daily diary study using ecological momentary assessment of self-reported stress, emotions, alcohol-related cravings, pain, and discomfort via a web-based survey, which was conducted 4 times daily. Participants also participated in structured interviews throughout the study to assess daily alcohol use and to validate self-reported and physiological stress markers. In the analysis, we first used existing artifact detection methods and physiological signal processing approaches to assess the quality of the physiological data. Next, we examined the descriptive statistics for self-reported outcomes. Finally, we investigated the associations between the features of physiological signals and self-reported outcomes.

**Results:**

We determined that 87.86% (1,032,265/1,174,898) of the EDA signals were clean. A comparison of the frequency of skin conductance responses per minute with previous research confirmed that the physiological signals collected in the ambulatory setting were successful. The results also indicated that the statistical features of the EDA and HRV measures were significantly correlated with the self-reported outcomes, including the number of stressful events marked on the sensor device, positive and negative emotions, and experienced pain and discomfort.

**Conclusions:**

The results demonstrated that the physiological data collected via an Empatica E4 wearable sensor device were consistent with previous literature in terms of the quality of the data and that features of these physiological signals were significantly associated with several self-reported outcomes among a sample of adults diagnosed with alcohol use disorder. These results suggest that ambulatory assessment of stress is feasible and can be used to develop tailored mobile health interventions to enhance sustained recovery from alcohol use disorder.

## Introduction

### Background

A well-established literature describes the important role of stress in addiction and the risk of relapse. For example, laboratory studies have shown that acute stressors increase drug-seeking behaviors in animals [1] and that physiological stress responses in laboratory situations predict relapse among humans [2]. There is also considerable overlap in the neural circuitry affected by stress and substance use [1]. Thus, the associations among cravings, negative emotions, and substance use have been described by a negative reinforcement model whereby the combination of craving, withdrawal-induced negative effect, and a dysregulated reward system during abstinence leads to increased vulnerability to relapse [1]. This model suggests that the ability to automatically detect moments of stress in real-world settings and deliver just-in-time tailored interventions can provide a powerful tool to prevent relapse, especially during the early stages of recovery when relapse risk is highest [3].

A common approach to monitoring stress is to analyze physiological signals, such as electroencephalography, blood volume pulse (BVP), heart rate variability (HRV), galvanic skin response, electrodermal activity (EDA), and electromyography [4-6]. In this study, we focus on assessing the quality of the measured EDA and HRV values as biomarkers of stress in human participants. These 2 signals have been identified as the most useful physiological signals for detecting stress in real-life, ambulatory settings [7]. EDA is one of the most direct methods for measuring the activation of the sympathetic nervous system induced by physical demands and mental stress. EDA measures the variation in the electrical conductance of the skin in response to sweat gland activity. The sympathetic nervous system controls sweat gland activity. If the sympathetic branch of the autonomic nervous system (ANS) is activated by physical demands or mental stress, the number of active sweat gland activity increases, which, in turn, increases skin conductance. Thus, higher levels of EDA are associated with increased levels of stress [8].

HRV refers to the variability of the time interval between consecutive heartbeats in individuals and can be computed from BVP signal readings. Previous research has established HRV as an objective measure of individual differences in emotional responses. In particular, HRV provides information about the flexibility of the ANS, the ease with which an individual can transition between high and low arousal states [9]. In general, higher HRV (or greater variability between the heartbeats) can mean that either the body has a strong ability to tolerate a current state of heightened stress or the body is recovering from previous accumulated stress. At rest, a higher HRV generally indicates a healthier state that shows greater resilience and flexibility in the ANS. In active states, relatively lower HRV might demonstrate better health conditions in individuals, as the heart adjusts to the increased demand [10].

To date, most research studies that have used physiological signals to detect stress have been conducted in controlled laboratory settings [7,11]. This research has demonstrated that multiple physiological signals and derived features can accurately detect induced stress using a variety of stimuli, such as Stroop color tests, mental arithmetic, or public speaking challenges [4]. One advantage of stress detection in these controlled environments is that most often, the *ground truth* of the condition is known (ie, stressed vs not stressed). However, induced stress in artificial laboratory settings may lack external assessment and thus may not represent the stress experienced by individuals in their daily lives [12]. As a result, recent efforts have used wearable sensors to provide continuous, ambulatory monitoring of stress in uncontrolled, real-world settings [7,11]. However, the potential of this nascent research is characterized by a number of challenges that limit its application. Among these gaps, most research has focused on the ambulatory assessment of stress among healthy adults; very little research has been conducted among clinical populations, such as adults diagnosed with alcohol use disorder (AUD). Furthermore, although previous research suggests that including additional information about the context of a stressful event can improve stress detection in ambulatory settings, this is not commonly achieved.

### Objectives

To address these gaps, this study used a multimodal approach to investigate the associations between 2 physiological signals (EDA and HRV) and self-reported outcomes, including alcohol use, heightened stress, positive and negative emotions, alcohol-related cravings, pain, and discomfort, among adults seeking treatment for AUD during a 2-week uncontrolled data collection. Specifically, the aims of this study are 3-fold: (1) to assess the quality of the physiological signals collected from an unobtrusive wearable sensor device, (2) to examine the associations between EDA and self-reported outcomes, and (3) to examine the associations between HRV and self-reported outcomes.

## Methods

### Participants and Procedures

A convenience sample of 11 participants (10 females) was recruited from adults seeking care at a mental health facility in a Western state in the United States. Potential participants were identified from 2 points in the consort flow of a larger study examining the effectiveness of contingency management treatment among adults with co-occurring serious mental illness and moderate to severe AUD. First, we recruited participants among those who did not meet the primary inclusion criteria of the larger study: Diagnostic and Statistical Manual of Mental Disorders, fifth edition (DSM-5) diagnosis of a serious mental illness or DSM-5 diagnosis of moderate to severe AUD. Participants were also identified from among those who did not meet the secondary eligibility criteria, after an induction phase and before randomization to the contingency management conditions. These individuals either failed to achieve an average urine ethyl glucuronide level that indicated recent heavy drinking (>349 ng/mL) or failed to attend at least one study visit during the last week of the 4-week induction phase.

Participants in this study met the following inclusion criteria: (1) aged 18-65 years and (2) self-reported consumption of 4 or more standard drinks on 5 or more occasions in the past 60 days. Participants were also required to own a smartphone with a data plan that allowed them to respond to the ecological momentary assessment (EMA) survey (described in the following section). Exclusion criteria included (1) current DSM-5 diagnosis of a severe drug use disorder, (2) inability to demonstrate competency to provide consent on the MacArthur Competence Assessment Tool for Clinical Research, (3) risk of medically dangerous alcohol withdrawal (ie, seizure within the last 12 months and concern by participant or clinician regarding a potentially dangerous withdrawal), (4) previous diagnosis of dementia, and (5) determination (by the principal investigator of the larger study) that participation would be medically or psychiatrically unsafe.

### Data Collection

This study included 3 components: (1) a daily diary study using EMAs of self-reported emotions, cravings, and stress via a web-based survey, prompted 4 times daily; (2) a wearable sensor device (Empatica E4 wristband) that captured continuous physiological markers of stress, including heart rate (HR), skin temperature, bodily movement, HRV, and skin conductance; and (3) structured qualitative interviews to assess daily alcohol use, using a timeline follow-back calendar, and to validate self-reported and physiological markers of stress.

### Measures

#### Continuous Monitoring of Physiological Stress

**Figure 1:**
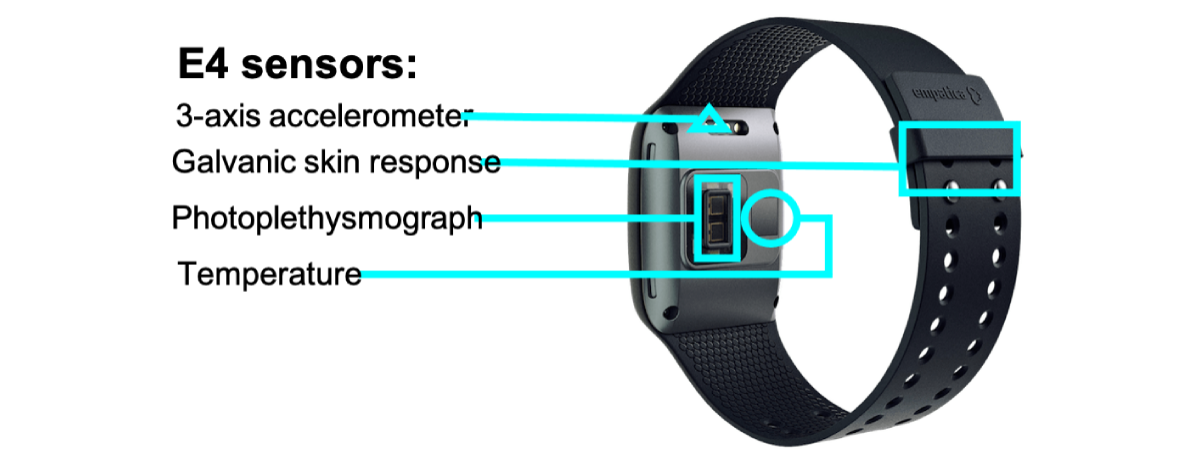
Empatica E4 sensor.

Each participant was asked to wear an Empatica E4 wristband to record continuous, real-time physiological measures of stress in their daily lives (Figure 1). The noninvasive E4 wristband is a wearable physiological sensor device that provides high-quality data that indicate arousal of the ANS (ie, stress). The E4 contains 4 sensors: (1) photoplethysmography to provide BVP, from which HR, HRV, interbeat interval (IBI), and other cardiovascular features may be derived; (2) EDA, used to measure sympathetic nervous system arousal and to derive features related to stress, engagement, and excitement; (3) a 3-axis accelerometer to capture motion-based activity; and (4) an infrared thermopile, used to measure skin temperature. Physiological data from these sensors were stored in the onboard memory of the E4 and downloaded by the research staff at each follow-up visit. The E4 weighs 40 g (1.41 oz) and is worn like a wristwatch, and all the sensors are embedded in the device. The E4 also includes a push-button interface that allows for data annotation. Previous research has assessed the validity of physiological signals recorded by an Empatica E4 device, such as EDA, HRV, and IBI, against the standard clinical ground truth [13,14]. Moreover, previous studies indicate that E4 is among the most commonly used physiological sensor devices in scientific research and validate its usefulness in detecting atrial fibrillation [15] and emotional arousal and stress [16,17].

The study staff provided instructions about proper handling and wear of the E4 wristband during a scheduled meeting after the individuals agreed to participate in the study. For example, participants were instructed to remove the wristband each night while sleeping and at other times during which the device may be damaged (eg, in the shower or bath) and to wear the device on the same wrist throughout the study. The training session also included instructions on how to use the *stress event marker button* on the E4 wristband. Participants were asked to press this button any time they felt *more stressed, overwhelmed, or anxious than usual*. These tag markers were summed for each participant to provide a daily tally of the number of perceived stress events.

#### EMA-Based Survey

Data collection for the EMA component was performed using a mobile phone–based survey. The survey assessed the perceptions of positive and negative emotions, alcohol-related cravings, and experiences of pain and discomfort. Participants responded to 4 timed signals throughout the day that corresponded to early morning (waking), noon, late afternoon, and bedtime for up to 14 consecutive days.

The measurement of *alcohol-related cravings* was derived from previous research [18,19] and included 3 items: (1) “[since last assessment], the idea of using alcohol has intruded upon my thoughts;” (2) “[since last assessment], I have missed the feeling alcohol can give me;” and (3) “[since last assessment], I have thought about how satisfying alcohol can be.” Response options included a 5-point scale, ranging from 1 (strongly disagree) to 5 (strongly agree). An aggregate measure of alcohol-related cravings was created by averaging the 3 items in each of the 4 assessments. An average daily alcohol-related craving score was then created for each participant for each day of the study.

*Positive and negative emotions* were assessed using items drawn from the extended version of the Positive and Negative Affect Scale (PANAS) [20]. Negative affect was assessed by asking, “[Since last assessment], have you felt [irritable/lonely/sad/guilty/ashamed/anxious/stressed]?” Similarly, participants reported their positive affect using the following terms: warmhearted, enthusiastic, affectionate, relaxed, calm, happy, joyful, and loving. Responses for each item were assessed on a 5-point scale, ranging from 1 (not at all) to 5 (extremely). Aggregate measures of daily negative and positive affect were created by first averaging across the respective items in each of the 4 assessments. Next, average positive and negative emotion scores were created for each participant on each day of the study.

Measurement of *pain and discomfort* was assessed with 2 items: (1) “[since last assessment], have you felt any physical discomfort?” and (2) “[since last assessment], have you felt any physical pain?” Five response options included 1=nonexistent, 2=slight, 3=moderate, 4=intense, and 5=unbearable. Average pain and discomfort scores were created for each participant on each day of the study.

#### Qualitative Debriefing Interview

Throughout the study, participants attended up to 6 short follow-up sessions to meet with study staff on an every-other-day basis (eg, Monday, Wednesday, and Friday). During the follow-up sessions, the study staff ascertained whether the participants were experiencing any problems or difficulties with either the wearable wristband device or completing the EMA surveys on their cell phones. The follow-up sessions also included administration of a timeline follow-back measure of recent alcohol use [21]. In this procedure, participants were first presented with a chart of the US Standard Drink definition and then asked to indicate the number of drinks consumed on each calendar day since the previous assessment. At each follow-up visit, participants exchanged their current E4 wristband device for a fully charged device with available onboard memory for new data collection.

### Statistical Analysis

#### Overview

We conducted a series of statistical analyses in 4 steps. First, we assessed the quality of the physiological signals, EDA and BVP. As described in the following sections, we used the recommended tools and procedures of the Empatica 4 guidelines to remove artifacts and extract features of the EDA and BVP signals for use in further analyses. Empatica E4 assesses the HR and IBI from the BVP signal using a proprietary algorithm [22]. Second, we examined descriptive statistics, including interitem correlations among patient-reported outcomes, aggregated at the day level. In the final 2 steps, we investigated the associations of the EDA signal (step 3) and the HRV signal (step 4) with the day-level self-reported outcomes.

#### EDA Quality Assessment

Physiological signals such as EDA are prone to noise and artifacts, especially when acquired in uncontrolled real-life scenarios. Therefore, we preprocessed the EDA signal to remove the most common artifacts, including environmental, sensor motion, and muscle movement artifacts. We used EDA Explorer public scripts to perform automatic artifact and noise detection [23]. During the process, a high-pass filter was first applied to smooth the raw EDA signals and remove low-frequency noise. Then, a multiclass classifier labeled the signal as clean, noisy, or questionable. The accelerometer and temperature data as well as the EDA were used in this process. Further analyses were performed using only the clean parts of the signal.

Trough-to-peak (TTP) and continuous decomposition analysis (CDA) are 2 commonly used analyses to assess the quality of EDA signals. We performed these analyses using the LedaLab toolbox (MATLAB program [MathWorks] suggested by the Empatica Manual for signal processing). In the TTP analysis, we set the sample rate to 1 Hz and the minimum amplitude threshold to 0.01 µS. In the CDA analysis, the EDA signal was decomposed into phasic and tonic components to increase the temporal precision. LedaLab provides information on the number of skin conductance responses (SCRs) and the SCR onset for each TTP and CDA analysis of the EDA signal. We downsampled the data from 4 Hz to 1 Hz and then computed the average SCRs per minute for all 11 participants to compare the results of the CDA and TTP analysis with previous research by plotting the frequency of the SCRs per minute [24].

**Figure 2:**
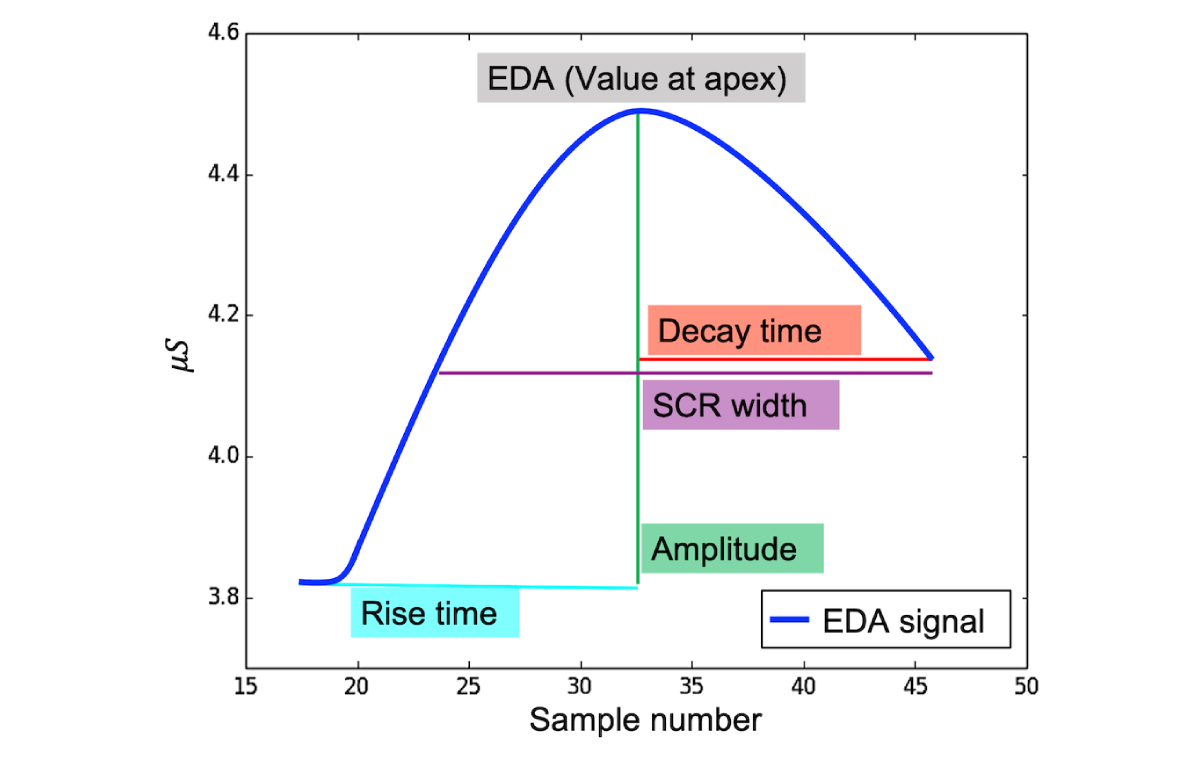
An example of an electrodermal activity peak. EDA: electrodermal activity; SCR: skin conductance response.

The next step was to extract the features that captured the patterns in the EDA signal. The EDA peak detection analysis provides a set of features corresponding to each EDA peak. We used EDA Explorer public scripts to detect the EDA peaks [23]. Previous studies have shown that peaks from EDA signals correlate with emotional arousal in humans. As shown in Figure 2, the values at apex, rise time, decay time, amplitude, and SCR width are standard features that can be extracted from the peaks of the EDA signal. Table 1 lists the features extracted from the EDA peaks.

**Table 1:** Description of the features that are extracted from the electrodermal activity peaks.

Feature	Description
EDA^a^	The EDA value at apex of the peak
Rise time	Time (microseconds) taken by the EDA peak to reach its maximum value
Maximum derivative	The maximum
Amplitude	The amplitude
Decay time	Time (microseconds) taken by the signal to drop from the apex to the minimum of the peak
Skin conductance response width	The width of the peak (number of the samples)
AUC^b^	2D area under the EDA peak curve

^a^EDA: electrodermal activity.

^b^AUC: area under the curve.

#### HR Quality Assessment

Measuring HR is a routine part of a clinical examination. The resting HR (RHR) of individuals reflects their overall health. We measured the RHR of the participant using IBI derived from the photoplethysmography sensor to identify any irregularities in the HR data. We note that the IBI data for this study were provided by Empatica and represent the time in milliseconds between two successive heartbeats (the R-R interval). This proprietary algorithm [22] removed incorrect peaks because of noise and artifacts in the BVP signal. Using the IBI sequence provided by Empatica, we extracted statistical features from the HR, including the mean value of the HR (MHR), minimum value of the HR (MNHR), maximum value of the HR (MXHR), and SD of the HR signal during the study.

Time domain analysis and frequency domain analysis are 2 standard methods for investigating HRV. In the time domain analysis, HRV measures were directly extracted from the IBI or RR interval signals. Frequency domain analysis extracts HRV measures from the power spectrum of the Fourier transform of the RR interval signals. In this study, we focus on the time domain HRV measures, including the mean of the RR interval (MRR), the SD of the RR interval (STDRR), the root mean square successive differences of the RR intervals (RMSSDs), and the coefficient of variance of the RR intervals (CVRR) [25,26].

## Results

### Demographics

The mean age of the participants was 40.27 years (SD 3.66; range 27-60 years). The majority of the sample was White, non-Hispanic (n=9). One participant identified as White, Hispanic, and one participant identified as American Indian or Alaskan Native. Of the 11 participants, 10 (91%) identified as female, and 1 (9%) participant identified as male.

### Data Set Quality Assessment

Previous research suggests that the collected physiological signals must maintain a number of quality measures to be considered valid for data analysis. For this research, we examined the quality of the collected data across several dimensions, including (1) the number of clean signals after artifact removal, (2) the distribution of the SCR values, using TTP and CDA analyses, and (3) the distribution of the HRV values. On the basis of the results of artifact detection, out of 1,174,898 EDA signal measurements, 1,032,265 (87.86%), 108,208 (9.21%), and 34,424 (2.93%) collected in total from all the participants were clean, noisy, and questionable, respectively. In both the TTP and CDA analyses, the EDA data were downsampled to a sampling rate of 1 Hz, and a minimum amplitude threshold of 0.01 µS was considered.

As expected, in both analyses, the SCRs per minute were positively skewed with most SCR values near or at 0.0 per minute. The results from the TTP analysis show a peak at 0 SCRs per minute, with a continuous decrease of up to 17.5 SCRs per minute. The results from the CDA analysis show a peak at 0 SCRs per minute, followed by a sharp decline at 1 SCRs per minute and later by a small increase from 15.0 to 17.5 SCRs. Thus, our data demonstrated results similar to those of a previous study that used the same analyses on 8 participants to assess the quality of EDA and HRV data collected with the E4 [24].

Table 2 reports the distribution of the HR averaged across low, normal, and high ranges separately for each participant. All the participants except participant 11 experienced similar distribution of HR during the data collection period. Among these 10 participants, 1.11% (59,982/5,399,250) of the study data were in the low range of 40-59 beats per minute (bpm), 82.76% (4,468,337/5,399,250) were in the normal range of 60-100 bpm, and 16.01% (4,468,337/5,399,250) were in the high range, above 100 bpm. However, for participant 11, only 44.87% (310,876/692,901) of the experienced HRs were in the normal range of 60-100 bpm, whereas more than half (379,119/692,901, 54.71%) of the HRs were in the high range of 101-200 bpm. On the basis of these results, we conclude that all the participants except participant 11 demonstrated normal HR distribution.

Table 3 reports the means and SDs for the HR features, including mean value the heart rate (MHR), MXHR, MNHR, and SD of the heart rate (STDHR) signal of the participants during the data collection. Likewise, Table 4 shows means and SDs for the heart variability measures for each participant during the entire data collection period.

**Table 2:** Total numbers and percentages of participants’ heart rate (bpm) across low, normal, and high ranges during the study period.

Subject	Total, N	Low (40–59 bpm^a^), n (%)	Normal (60-100 bpm), n (%)	High (101-200 bpm), n (%)
Participant 1	402,257	3626 (0.91)	290,316 (72.17)	108,267 (26.91)
Participant 2	715,794	9599 (1.34)	605,916 (84.65)	100,146 (13.99)
Participant 3	473,283	2601 (0.55)	386,090 (81.58)	84,554 (17.87)
Participant 4	606,614	15,592 (2.57)	533,903 (88.01)	50,999 (8.41)
Participant 5	738,475	953 (0.13)	555,673 (75.25)	181,685 (24.6)
Participant 6	650,860	14,739 (2.26)	551,797 (84.78)	84,276 (12.95)
Participant 7	340,036	399 (0.12)	263,814 (77.58)	75,801 (22.29)
Participant 8	388,833	6394 (1.64)	340,905 (87.67)	41,496 (10.67)
Participant 9	535,352	3592 (0.67)	467,507 (87.33)	64,213 (11.99)
Participant 10	547,746	2487 (0.45)	472,416 (86.25)	72,789 (13.29)
Participant 11	692,901	2164 (0.31)	310,876 (44.87)	379,119 (54.71)

^a^bpm: beats per minute.

**Table 3:** Means and SDs for the statistical features extracted from the heart rate signal.

Subject	Mean value of the heart rate, mean (SD)	Minimum value of the heart rate, mean (SD)	Maximum value of the heart rate, mean (SD)	SD of the heart rate, mean (SD)
Participant 1	104.72 (9.81)	51.17 (19.27)	167.32 (17.9)	10.21 (1.74)
Participant 2	87.05 (6.15)	45.08 (5.01)	186.11 (14.61)	12.1 (2.86)
Participant 3	88.83 (5.29)	51.26 (6.18)	182.97 (14.71)	11.28 (3.15)
Participant 4	71.27 (6.15)	35.64 (6.15)	159.71 (27.55)	9.21 (2.90)
Participant 5	86.3 (8.03)	45.92 (11.18)	171.66 (21.96)	9.85 (2.27)
Participant 6	74.11 (7.88)	37.40 (4.73)	185.45 (12.29)	14.97 (3.94)
Participant 7	91.07 (7.84)	45.48 (5.06)	168.99 (19.72)	11.72 (2.91)
Participant 8	78.54 (9.48)	38.53 (4.52)	180.56 (12.34)	10.37 (2.23)
Participant 9	79.04 (6.88)	48.22 (4.28)	169.47 (30.22)	9.87 (1.54)
Participant 10	84.79 (3.38)	42.88 (5.47)	166.88 (13.08)	12.54 (0.98)
Participant 11	95.19 (8.14)	44.29 (9.58)	180.7 (29.88)	22.53 (8.45)

**Table 4:** Means and SDs for the statistical features extracted from the heart rate variability measures.

Subject	Mean value of all of the RR intervals, mean (SD)	SD of the RR interval, mean (SD)	Root mean square, mean (SD)	Covariance of SD, mean (SD)	Covariance of all the RR intervals, mean (SD)
Participant 1	0.58 (0.05)	0.06 (0.01)	0.06 (0.01)	0.11 (0.02)	0.10 (0.02)
Participant 2	0.70 (0.05)	0.00 (0.02)	0.07 (0.02)	0.10 (0.02)	0.13 (0.02)
Participant 3	0.69 (0.04)	0.08 (0.02)	0.07 (0.02)	0.11 (0.03)	0.12 (0.02)
Participant 4	0.86 (0.07)	0.10 (0.01)	0.07 (0.01)	0.08 (0.02)	0.12 (0.03)
Participant 5	0.71 (0.06)	0.08 (0.01)	0.07 (0.01)	0.10 (0.03)	0.11 (0.02)
Participant 6	0.84 (0.08)	0.13 (0.03)	0.11 (0.02)	0.13 (0.03)	0.15 (0.05)
Participant 7	0.68 (0.06)	0.09 (0.02)	0.07 (0.02)	0.10 (0.03)	0.13 (0.03)
Participant 8	0.78 (0.10)	0.10 (0.02)	0.09 (0.02)	0.11 (0.01)	0.12 (0.03)
Participant 9	0.78 (0.08)	0.09 (0.01)	0.07 (0.01)	0.09 (0.02)	0.12 (0.01)
Participant 10	0.72 (0.03)	0.10 (0.01)	0.09 (0.01)	0.13 (0.02)	0.14 (0.01)
Participant 11	0.68 (0.05)	0.13 (0.01)	0.09 (0.01)	0.13 (0.02)	0.19 (0.06)

### Self-reported Outcomes: Descriptive Statistics and Bivariate Correlations

Table 5 reports the descriptive statistics for the self-reported outcomes, computed at the daily level, including mean, SD, median, minimum and maximum, and 1st and 3rd quartiles. Overall, the participants reported a mean of 3 stressful events each day (SD 2.9), with a range of 0-17 stressful moments per day. Participants reported consuming between 0 and 18 servings of alcohol daily, with a mean of 2.8 servings per day (SD 5.2). Mean values for self-reported alcohol cravings, positive and negative emotions, and pain and discomfort ranged from 2.2-2.9, with values ranging between 1 and 5.

**Table 5:** Descriptive statistics of the self-reported outcomes.

Self-reported outcomes	Mean (SD)	Median	Minimum	Maximum	1^st^ quartile	3^rd^ quartile
Stress events	3 (2.9)	2	0	17	1	3.5
Alcohol use	2.8 (5.2)	0.0	0.0	18.0	0.0	3.0
Alcohol cravings	2.9 (0.9)	3.0	1.0	4.8	2.3	3.5
Positive emotion	2.5 (0.6)	2.5	1.3	3.9	2.1	2.9
Negative emotion	2.3 (0.8)	2.5	1.0	2.9	1.5	2.9
Discomfort	2.3 (1.0)	2.3	1.0	5.0	1.0	3.0
Pain	2.2 (1.1)	2.5	1.0	5.0	1.0	3.0

Table 6 shows the results of the correlation analysis of the self-reported outcome variables. The intersection of a pair of outcomes on the left side of the diagonal displays the correlation coefficient, indicating the strength of the relationship between them. As seen in Table 6, the number of stress events was significantly and positively associated with self-reported negative mood (*r*=0.21; *P*=.002) and pain (*r*=0.18; *P*=.006). Self-reported alcohol use was significantly and positively correlated with self-reported cravings (*r*=0.46; *P*<.001) and negatively correlated with self-reported negative mood, discomfort, and pain, with coefficient correlation values of −0.38, −0.44, and −0.45, respectively (all *P* values <.001). Self-reported alcohol-related cravings were also negatively correlated with days characterized by pain (*r*=−0.20; *P*=.005) and discomfort (*r*=−0.25; *P*=.007). Participant self-reports of discomfort and pain were significantly and positively correlated (*r*=0.93; *P*<.001). Each of these 2 measures was also significantly and positively correlated with participant self-reports of negative mood, with coefficient correlation values of 0.49 and 0.48 (both *P* values <.001)

**Table 6:** Bivariate correlation coefficient values of the self-reported outcomes.

Self-reported outcomes.	Stress events	Alcohol use	Alcohol cravings	Positive emotion	Negative emotion	Discomfort	Pain
Stress events	—^a^	—	—	—	—	—	—
Alcohol use	−0.11	—	—	—	—	—	—
Alcohol cravings	−0.001	0.46	—	—	—	—	—
Positive emotion	0.07	0.001	−0.16	—	—	—	—
Negative emotion	0.21	−0.38	0.02	−0.15	—	—	—
Discomfort	0.04	−0.44	−0.20	−0.02	0.49	—	—
Pain	0.18	−0.45	−0.25	−0.05	0.48	0.93	—

^a^Not applicable.

### Correlations Among EDA and Patient-Reported Outcomes

Table 7 shows the Spearman correlation coefficients between the EDA features and the daily aggregated self-reported outcomes. As seen in the table, the number of stress events was positively associated with the amplitude (*r*=0.26; *P*=.005) and counts (*r*=0.27; *P*=.003) of the EDA peaks. In contrast, the number of stress events was negatively correlated with the decay time (*r*=−0.20; *P*=.03), SCR width (*r*=−0.34; *P*<.001), and area under the curve (*r*=−0.32; *P*<.001) of the EDA peaks. These results suggest that participants experienced more and higher peaks in the EDA signal on days characterized by more stress and that the peaks in the EDA signal tended to drop more rapidly—and were narrower—during more stressful days. Among the other self-reported outcomes, the number of alcohol drinks was positively associated with the decay time of the EDA peaks (*r*=0.18; *P*=.08). Daily averages of positive mood were positively associated with EDA rise time (*r*=0.23; *P*=.02), amplitude (*r*=0.20; *P*=.06), and decay time (*r*=0.17; *P*=.09). Rise time (*r*=0.28; *P*=.005) and amplitude (*r*=0.41; *P*<.001) were also positively associated with aggregate levels of self-reported negative mood. In contrast, the decay time of the EDA signal was negatively correlated with the daily levels of discomfort (*r*=−0.18; *P*=.08) and pain (*r*=−0.22; *P*=.03).

**Table 7:** Spearman correlation coefficients between the daily electrodermal activity features and the daily self-reported outcomes.

Feature	Self-reported outcome						
	Stress	Alcohol	Cravings	Positive emotion	Negative emotion	Discomfort	Pain
Electrodermal activity	−0.09	−0.03	0.01	−0.14	0.17	−0.06	−0.07
Rise time	0.11	−0.06	0.10	0.23^a^	0.28^b^	−0.12	−0.12
Maximum derivative	0.04	0.01	0.16	0.09	0.11	−0.07	−0.07
Amplitude	0.26^b^	−0.02	0.16	0.20^c^	0.41^d^	0.01	0.01
Decay time	−0.20^a^	0.18^c^	0.12	0.17^c^	−0.10	−0.18^c^	−0.22^a^
Skin conductance response width	−0.34^d^	0.06	0.15	−0.01	0.02	−0.12	−0.14
Area under the curve	−0.32^d^	0.04	0.17	0.01	0.05	−0.07	−0.08
Counts	0.27^b^	0.05	0.09	0.09	0.09	0.10	0.10

^a^Significance code .05.

^b^Significance code .01.

^c^Significance code .10.

^d^Significance code .001.

### Correlations Among HRV and Self-reported Outcomes

Table 8 displays the Spearman correlation coefficients for the associations between HRV measures and self-reported outcomes. With the exception of the RMSSD and covariance of SD (CVSD), all the HRV features were positively associated with the number of stress reported at the daily level: MRR (*r*=0.22; *P*=.02), STDRR (*r*=0.22; *P*=.02), CVRR (*r*=0.27; *P*=.004), MHR (*r*=0.28; *P*=.002), and STDHR (*r*=0.21; *P*=.02). Similarly, almost all the HRV features were positively correlated with aggregate levels of self-reported positive mood: MRR (*r*=0.26, *P*=.01), STDRR (*r*=0.21; *P*=.04), RMSSD (*r*=0.26; *P*=.01), CVSD (*r*=0.24; *P*=.02), CVRR (*r*=0.20, *P*=.049), and MHR (*r*=0.26; *P*=.009). All but one of the HRV features was positively correlated with aggregate levels of self-reported negative mood: MRR (*r*=0.18; *P*=.08), STDRR (*r*=0.22; *P*=.03), CVSD (*r*=0.22; *P*=.03), CVRR (*r*=0.29; *P*=.005), MHR (*r*=0.33; *P*=.001), and STDHR (*r*=0.32; *P*=.002). The MRR was negatively associated with aggregated levels of self-reported discomfort (*r*=−0.21; *P*=.04) and pain (*r*=−0.18; *P*=.08). The MHR was also negatively associated with aggregated levels of self-reported discomfort (*r*=−0.22; *P*=.003) and pain (*r*=−0.20; *P*=.05). No significant associations were found between HRV features and self-reported alcohol use or alcohol-related cravings.

**Table 8:** Spearman correlation coefficients between the daily heart rate variability measures and the daily self-reported outcomes.

Feature	Self-reported outcome						
	Stress	Alcohol	Cravings	Positive	Negative	Discomfort	Pain
Mean value of all the RR intervals	0.22^a^	−0.07	0.00	0.26^a^	0.18^b^	−0.21^a^	−0.18^b^
SD of the RR interval	0.22^a^	−0.07	0.04	0.21^a^	0.22^a^	−0.06	−0.06
Root mean squared SD	0.08	−0.04	0.02	0.26^a^	0.15	−0.10	−0.12
Covariance of SD	0.12	0.00	0.05	0.24^a^	0.22^a^	−0.10	−0.13
Covariance of all the RR intervals	0.27^c^	−0.05	0.04	0.20^a^	0.29^c^	−0.08	−0.08
Mean value of heart rate	0.28^c^	0.01	0.03	0.26^c^	0.33^d^	−0.22^a^	−0.20^b^
SD of the heart rate	0.21^a^	−0.02	0.04	0.14	0.32^c^	−0.07	−0.07

^a^Significance code .05.

^b^Significance code .10.

^c^Significance code .01.

^d^Significance code .001.

## Discussion

### Principal Findings

The overall goal of this study is to examine the feasibility of using 2 physiological markers of stress, EDA and HRV, obtained from an unobtrusive wristband device in an ambulatory setting, as a first step toward developing mobile health (mHealth) interventions to help prevent alcohol relapse. To achieve this goal, we first assessed the quality of the continuous monitoring of physiological signals using an Empatica E4 wearable sensor device. We determined that 87.88% (1,032,265/1,174,898) of the EDA signals were clean, whereas only 9.21% (108,208/1,174,898) of the EDA signals could be considered noise. A comparison of the distribution of the EDA SCRs also demonstrated high correspondence between our study and previous studies that used similar techniques [24]. We also noted that the distribution of HR signals for 10 of the 11 participants was within a typical distribution. On the basis of these results, we can conclude that physiological data met the established quality criteria.

In the next steps, we examined associations between features of the EDA and HRV signals with a number of self-reported outcomes, including daily tallies of stress events and alcoholic drinks as well as aggregated levels of alcohol-related cravings, positive and negative moods, and experiences of pain and discomfort. In total, 2 features of the EDA signal (amplitude and peak counts) were positively associated with the number of stress events reported each day, whereas 3 features (decay time, SCR width, and area under the curve) were negatively associated with daily tallies of stress events. These results were as expected and suggest that participants experienced more and higher EDA peaks on days of heightened stress and that the EDA signal varied more rapidly during more stressful intervals. The results also showed that some (but not all) EDA features were positively associated with self-reported positive and negative emotions. In general, EDA features were not significantly correlated with the daily use of alcohol or alcohol-related cravings or with experiences of pain or discomfort.

However, almost all HRV features were significantly and positively associated with daily tallies of stress events. These results were as expected and indicated that participants experienced greater HRV on days characterized by higher levels of stress. Nearly all HRV features were also significantly and positively associated with the aggregate levels of positive and negative emotions. Thus, the results suggest an increased recovery ability of the participants in stressful situations or when feeling excessive positive or negative moods. Similar to the findings for the EDA features, HRV features were generally not significantly associated with daily use of alcohol or alcohol-related cravings or experiences of pain or discomfort.

### Limitations

This study was designed to establish the feasibility of assessing physiological data in an ambulatory setting to inform the development of a future mHealth intervention. Thus, the major limitation of this pilot study is the small sample size, which limited our ability to test hypotheses regarding associations between physiological signals and self-reported outcomes with full statistical power. Future studies that include larger and more representative samples are needed to replicate our findings. We also note that our analyses did not account for demographic characteristics of the participants, such as age, personality characteristics, race, or gender. Future work that examines how these and other demographic characteristics might play a role in these processes is needed.

We further acknowledge that although the uncontrolled nature of the data collection in ambulatory settings was a strength of this study, this may have introduced some subjective bias into the assessment of stress events. Future research in ambulatory settings should also consider how HRV is affected by sleep and circadian processes [27] as well as by exercise or simple ambulatory activities [28] such as walking, which our analyses did not take into account. Finally, one reason for the lack of strong associations among the physiological signals and some of the self-reported outcomes may be attributed to the single-level correlation analyses used in this study. This approach did not take into account the nested nature of the data: 4 prompts each day, nested within multiple days, and nested within 11 participants. More sophisticated multilevel modeling of these associations that takes such clustering into account might provide additional insights about within-person associations as well as between-person differences in these effects.

### Conclusions

We investigated the associations of physiological signals, EDA and HRV, with self-reported outcomes among adults diagnosed with AUD in a 14-day uncontrolled data collection. The results demonstrated that the physiological data collected via an Empatica E4 wearable sensor device were useful and that features of these physiological signals were significantly associated with several self-reported outcomes, including identification of stress events, daily use of alcohol, negative and positive emotions, and pain and discomfort. Future research is needed to further validate these findings to develop tailored mHealth interventions to enhance sustained recovery from AUD.

## Abbreviations

ANS: autonomic nervous system
AUD: alcohol use disorder
BPM: beats per minute
BVP: blood volume pulse
CDA: continuous decomposition analysis
CVRR: coefficient of variance of the RR intervals
CVSD: covariance of SD
EDA: electrodermal activity
EMA: ecological momentary assessment
HR: heart rate
HRV: heart rate variability
IBI: interbeat interval
mHealth: mobile health
MHR: mean value of the heart rate
MNHR: minimum value of the heart rate
MRR: mean of the RR interval
MXHR: maximum value of the heart rate
PANAS: Positive and Negative Affect Scale
RMSSD: root mean square successive differences of the RR interval
SCR: skin conductance response
STDRR: SD of the RR interval
TTP: trough-to-peak
